# Students' benefits and barriers to mental health help-seeking

**DOI:** 10.1080/21642850.2014.963586

**Published:** 2014-10-24

**Authors:** Rebecca A. Vidourek, Keith A. King, Laura A. Nabors, Ashley L. Merianos

**Affiliations:** ^a^ Health Promotion and Education, University of Cincinnati, PO Box 210068, ML 0068, 526TC, Cincinnati, OH45221, USA; ^b^ Counseling, University of Cincinnati, Cincinnati, OH45221, USA

**Keywords:** mental health, stigma, college student, barriers, benefits

## Abstract

Stigma is recognized as a potential barrier to seeking help for a mental health disorder. The present study assessed college students' perceived benefits and barriers to obtaining mental health treatment and stigma-related attitudes via a four-page survey. A total of 682 students at one Midwestern university participated in the study. Findings indicated that females perceived a greater number of benefits to having participated in mental health services and held significantly lower stigma-related attitudes than did males. Students who had ever received mental health services reported significantly more barriers to treatment than did students who had never received services. Health professionals should target students with educational programs about positive outcomes related to receiving mental health services and work with treatment centers to reduce barriers for receiving services.

## 1. Background

Mental health problems are a significant issue among university students. Approximately 32% of university students report having mental health problems (Eisenberg, Hunt, Speer, & Zivin, 2011). Numerous mental health issues are experienced by college students and include anxiety, depression, suicidal ideation, and overwhelming stress (American College Health Association, 2009). According to the American College Health Association-National College Health Assessment (ACHA-NCHA), 3 in 10 college students reported difficulty functioning due to feeling depressed (American College Health Association, 2009). In addition, approximately 6% of college students seriously considered suicide and 1% attempted suicide in the past year.

Individuals diagnosed with mental health disorders often find relief from symptoms by participating in treatment (National Alliance on Mental Illness, 2014). Psychotherapy, which includes investigating thoughts, feelings, and behaviors, is one treatment available to individuals with mental health disorders (Mental Health America, 2014). In combination with medication, psychotherapy is recognized as an effective treatment for mental health disorders. However, many college students do not seek treatment for mental health disorders as they feel that symptoms are typical of college stress and also express concern that others will judge them for seeking treatment (Eisenberg, Golberstein, & Gollust, 2007).

Not surprisingly, only one-third of students with mental health disorders have participated in mental health treatment (Eisenberg et al., 2011). Without treatment, students experiencing mental health disorders are at high risk for lower grade point averages, school dropout, and unemployment (United States Government Accountability Office, 2008). A greater understanding of the barriers and benefits to participation in mental health services among college students will inform service providers who need to help students feel comfortable in seeking professional help when they experience mental health problems (Pietruszka, 2007).

With this in mind, the Health Belief Model is often used to examine factors associated with health behaviors (Booth, McKenzie, Stone, & Welk, 1998). For the purpose of the present study, perceived benefits and barriers to mental health help-seeking are being explored. Previous research found that perceived barriers have a significant effect on college students' health behavior choices (Von Ah, Ebert, Ngamvitroj, Park & Kang, 2003). Perceived benefits and barriers to help-seeking were specifically selected due to their influence on decision-making and ultimately action (Glanz, Rimer, & Su, 2005). Identifying both perceived benefits and barriers may be useful to college health professionals developing mental health prevention and intervention programs.

### 1.1. Barriers and facilitating factors for seeking treatment

Barriers to mental health help-seeking reduce the likelihood individuals will seek treatment for a mental health disorder (Edlund, Unutzer, & Curran, 2006; Mojtabai et al., 2011). Research has identified barriers to mental health services such as lack of perceived need, perceived ineffectiveness of treatment, problems with access to care, and inconvenience (Bayer & Peay, 1997; Mojtabai, 2005; Sareen et al., 2007). As a group, university students may stigmatize others who experience mental illness (Gonzalez, Alegria, & Prihoda, 2005; Mowbray et al., 2006; Zivin, Eisenberg, Gollust, & Golberstein, 2009). Stigma, defined as negative attitudes held by others, is a barrier to receiving treatment for mental health disorders (Corrigan, Markowitz, Watson, Rowan, & Kubiak, 2003). Perceiving negative attributes such as believing individuals with mental health problems are weak, incompetent, and cannot take care of themselves, are common forms of stigma and may contribute to increases in harmful attitudes (Corrigan, Edwards, Green, Diwan, & Penn, 2001; Read & Law, 1999). In addition, negative attitudes may lead to discriminatory behaviors toward individuals with mental health problems as well as social isolation (Corrigan et al., 2001; Link, Yang, Phelan, & Collins, 2004). Unfortunately, people facing stigmatized attitudes may come to internalize negative views of the self and experience feelings of shame (Antonak & Livneh, 2000; Byrne, 2000). Negative views of treatment and shame related to experiencing mental illness may preclude young adults from seeking needed treatment (Byrne, 2000; Eisenberg et al., 2007; Gonzalez et al., 2005). However, some studies have not found this type of relationship (Golberstein, Eisenberg, & Gollust, 2009), indicating that further study of attitudes is needed. The present study seeks to examine whether or not stigma serves as a barrier to treatment among college students.

Eisenberg et al. (2011) proposed that skepticism about treatment efficacy is another barrier to participating in treatment. Study findings revealed that college students endorsed several barriers to participating in treatment. Among these were: (1) preferring to deal with mental health problems themselves, (2) not having enough time to participate in treatment, (3) questions about whether mental health treatment is effective in remediating problems, (4) a belief that stress is normal or the problem will get better without treatment, (5) lack of money, and (6) worry about what others would think if they found out about therapy participation. Mowbray et al. (2006) reported similar variables as barriers to seeking treatment and also found that a mistrust of providers may impede students from seeking help. Staff in campus mental health centers may be perceived as unfriendly, and long wait times for services may be “off-putting” for students.

Factors facilitating more positive attitudes are often at the opposite pole of those factors identified as barriers. Beyond this, believing that mental health treatment is effective and results in better health and social outcomes is one of the most critical factors influencing positive beliefs about mental health services and people with mental illness (Eisenberg et al., 2011; Mowbray et al., 2006). College students' perceived benefits of treatment are understudied. Identifying such benefits may aid college students in increasing help-seeking for mental health treatment.

### 1.2 Perceiver characteristics: relations with attitudes toward mental health services

Several characteristics of those making judgments, the “perceivers”, may influence college students' attitudes toward mental health services as well as their perceptions of barriers and benefits related to seeking treatment. For the current study, several perceiver characteristics were of interest, such as gender (Chandra & Minkovitz, 2006), ethnic group (Chamberlain et al., 2000), age (Read & Law, 1999), experience with family or friends with mental illnesses (Eisenberg et al., 2011), and involvement in campus life and activities (e.g. references). The current study assessed the influence of the aforementioned perceiver characteristics on attitudes toward individuals with mental health problems as well as the relationship of these factors with the number of perceived benefits and barriers to receiving treatment. Few researchers have examined the association among perceiver characteristics including sex, ethnicity, and experience with mental illness and perceived barriers and benefits to participating in mental health treatment, which is a contribution of the current study. Such information may be useful to college health professionals in increasing treatment rates and tailoring educational programs.

### 1.3. Purpose statement

The purpose of the present study is to examine benefits and barriers to mental health help-seeking behaviors among college students. More specifically, the following research questions were examined: (1) What are perceived benefits and barriers to mental health help-seeking behaviors among college students? (2) Do college students hold negative or stigmatized attitudes toward receiving services? (3) Do perceived benefits, barriers, and stigma-related attitudes differ based on: (a) sex, (b) grade, (c) race/ethnicity, (d) involvement in a campus organization, and (e) having a friend or family member with a mental health disorder.

## 2. Methods

### 2.1. Participants

Participants in the present study (*N* = 698) were English-speaking students enrolled in general education, health, fitness, and leisure classes at a Midwestern university in the USA. Sample size was calculated a priori. A sample of 379 was needed to have a confidence level of 95% with a confidence interval of 5%. Participation was strictly voluntary and no incentives were offered.

### 2.2. Instrumentation

After a comprehensive review of the literature, a survey was developed to examine college students' perceived benefits and barriers to mental health help-seeking behaviors. In order to incorporate components from The Health Belief Model, a new survey was developed to assess mental health help-seeking among college students. To establish face validity, the survey was developed based on a comprehensive review of the professional literature and individual discussions with college students, mental health professionals, and the research team (Bornstein, 2004). Suggestions were offered from these individuals regarding potential benefits and barriers to seeking help. To establish content validity, the survey was distributed to a panel of experts in health education, mental health, and survey research (Sireci, 2007). Suggested revisions and recommendations offered by the experts were incorporated into the final survey instrument. To establish stability reliability, the survey was distributed on two separate occasions (seven days apart) to a convenience sample of students (*N* = 25) (Feder, 2008). Pearson correlation coefficients were computed for the parametric subscales and yielded coefficients >0.85.

The survey was comprised of the following sections: Section 1 (benefits to help-seeking) assessed college students' perceived benefits to seeking treatment for a mental health problem (*n* = 14 items). This section required students to check all that apply by checking the appropriate boxes. Section 2 (barriers to help-seeking) assessed college students' perceived barriers to seeking treatment for a mental health problem (*n* = 14 items) and required students to check all that apply. Section 3 (stigma-related attitudes) examined perceived stigma to mental health disorders and treatment (*n* = 6 items) and required students to respond by using a 5-point Likert-type scale (1 = strongly disagree and 5 = strongly agree). Section 4 (background and demographics) required students to provide demographic and background information (*n* = 11 items) by filling in the blanks and checking the appropriate boxes.

### 2.3. Procedures

After obtaining consent to conduct the present study from the institutional review board, a convenience sample of students was obtained from college classes. Specifically, surveys were distributed to students in general education courses during regularly scheduled class times. Students reviewed an information form explaining the purpose of the study and the voluntary nature of participation. No names or personally identifying information was obtained on the information or survey; thus, students were assured that data were de-identified and that their participation was confidential. Students were also informed that they could withdraw from the study at any time should the survey make them uneasy or uncomfortable. All students presented with the survey elected to complete the survey.

### 2.4. Data analysis

All data analyses were performed using the Statistical Package for the Social Sciences (SPSS), Version 18.0. Frequency distributions, means, standard deviations, and ranges of scores were used to describe the demographic and background characteristics of participants. A series of analysis of variance (ANOVA) were performed to determine whether the number of benefits and the number of barriers differed based on sex, grade, involvement in a campus organization, and having a friend or family member with a mental health disorder. A series of multivariate analysis of variance (MANOVA) were performed to determine if stigma-related attitudes differed based on sex, grade, involvement in a campus organization, having a friend or family member with a mental health disorder, and the number of benefits and barriers.

## 3. Results

### 3.1. Demographic characteristics

A total of 682 students participated in the study. Of participants, 62.3% were female, whereas 37.7% of participants were male (Table 1). Concerning grade level, 37.6% were freshman, 26.0% were sophomores, 17.8% were juniors, 17.6% were seniors, and 1.0% were graduate students. The sample was predominantly Caucasian (77.8%) followed by African-American (12.9%), Asian (4.5%), and Hispanic (1.0%). A total of 3.7% of students self-identified as other race/ethnicity. Regarding living location, 40.5% reported living off campus. One in three (34.6%) reported living on campus and one in four (23.3%) reported living with parents. Nearly half of students were involved in campus organizations and 1 in 10 reported being in a fraternity or sorority. More than one-third of students (38.1%) reported that they had a family member or friend with a diagnosed mental health disorder.

**Table 1.** Demographic and background characteristics of participants.

Item	*n*	%
Sex		
Female	422	37.7
Male	255	62.3
Grade		
Freshman	254	37.6
Sophomore	176	26.0
Junior	120	17.8
Senior	119	17.6
Graduate student	7	1.0
Race/ethnicity		
White	523	77.8
African-American	87	12.9
Asian	30	4.5
Hispanic	7	1.0
Other	25	3.7
Fraternity or sorority member		
No	599	88.6
Yes	77	11.4
Involved in campus organizations		
No	370	54.9
Yes	304	45.1
Have ever visited a counselor for a mental health problem		
No	552	81.4
Yes	126	18.6
Has a friend or family member with a diagnosed mental health disorder		
No	415	61.9
Yes	255	38.1

Note: All categories do not total 698 due to missing data; percent refers to valid percent; and missing values are excluded.

### 3.2. Benefits of help-seeking

Results indicated that the top three perceived benefits were improved mental health, reduced stress, and resolving one's problems (Table 2). The lowest perceived benefits were increased energy, improved sleep, and increased social support.

**Table 2.** College students' perceived benefits and barriers tohelp-seeking for mental health problems.

	*n*	%
*Which of the following do you feel is a benefit of individuals seeking help for mental health problems?*		
Improved mental health	610	89.4
Reduced stress	591	86.7
Resolving one's problems	575	84.3
Self-awareness/personal growth	564	82.7
Happiness	555	81.4
Improved life satisfaction	546	80.1
Increased relationships	533	78.2
More optimistic attitude	532	78.0
Increased self-confidence	530	77.7
Increased communication	490	72.0
Increased comfort sharing feelings with others	485	71.1
Increased social support	458	67.2
Improved sleep	422	61.9
Increased energy	416	61.0
*Which of the following do you feel is a barrier for individuals seeking help for mental health problems?*		
Embarrassment	619	90.8
Denial that there is a problem	595	87.2
Not wanting to be labeled as “crazy”	496	72.7
Not knowing where to go for help	483	70.8
Not feeling comfortable sharing feelings with another person	478	70.1
Not wanting to talk to a counselor about personal issues	462	67.7
Wanting to handle problems on one's own	457	67.0
Cost	423	62.0
Fear of counselors	381	55.9
Not wanting to be admitted to a hospital	377	55.3
Lack of social support	330	48.4
Not wanting to be placed on medication	324	47.5
Not wanting help	321	47.1
Lack of insurance	274	40.2

Note: *n* = 698; Percent refers to valid percent; and missing values are excluded.

A series of one-way ANOVAs were conducted to determine if benefits of help-seeking differed based on demographic and background characteristics. Results indicated that females perceived significantly more benefits than males to seeking help for a mental health disorder (*F* = 5.342, *p* = .021). In addition, students with a family member or friend with a mental health disorder perceived a significantly higher number of benefits than students who did not have a family member or friend with a mental health disorder. Concerning race/ethnicity, white students perceived significantly more benefits to help-seeking than did non-white students. Results indicated no significant differences in perceived benefits between freshman/sophomores and juniors/seniors/graduate students and between students involved in a campus organization and those not involved in a campus organization. Lastly, results indicated no significant difference in perceived benefits based on ever visiting a mental health counselor.

### 3.3. Barriers to help-seeking

Results indicated that the top three perceived barriers were embarrassment, denial, and not wanting to be labeled crazy (Table 3). The lowest perceived barriers were lack of insurance, not wanting help, and not wanting to be placed on medication.

**Table 3.** College students’ perceived benefits and barriers to mental health help-seeking based on demographic and background characteristics.

Independent variable	Number of perceived barriers *M* (SD)	*F*	*p*
Sex			
Male	10.32 (3.77)	5.342	.021
Female	10.96 (3.30)		
Grade level			
Freshman/sophomore	10.87 (3.40)	2.348	.126
Junior/senior/graduate student	10.44 (3.65)		
Involved in campus organization			
No	10.60 (3.65)	10.596	.353
Yes	10.85 (3.30)		
Friend/family member diagnosed with mental health disorder			
No	10.43 (3.51)	6.923	.009
Yes	11.16 (3.43)		
Race/ethnicity			
Non-white	9.87 (3.54)	11.067	.001
White	10.95 (3.46)		
Independent variable	Number of perceived benefits *M* (SD)	*F*	*p*
Sex			
Male	8.64 (3.08)	1.76	.185
Female	8.95 (2.85)		
Grade level			
Freshman/sophomore	9.03 (2.80)	5.752	.017
Junior/senior/graduate student	8.46 (3.13)		
Involved in campus organization			
No	8.80 (3.03)	0.073	.787
Yes	8.86 (2.84)		
Friend/family member diagnosed with mental health disorder			
No	8.65 (2.95)	3.070	.080
Yes	9.06 (2.88)		
Race/ethnicity			
Non-white	8.11 (2.81)	11.234	.001
White	9.02 (2.95)		

Note: *n* = 698; Missing values are excluded.

A series of one-way ANOVA were computed to determine if perceived barriers to help-seeking differed based on demographic and background characteristics. Results found that freshmen/sophomores perceived a significantly greater number of barriers than did juniors/seniors/graduate students. Regarding race/ethnicity, white students perceived significantly more barriers to help-seeking than did non-white students. Additionally, students who had ever received mental health counseling perceived significantly more barriers to help-seeking than those who had not received mental health counseling. Results indicated no significant differences in perceived barriers between males and females, between students involved in a campus organization and those not involved, and between students with or without a family member or friend with a mental health disorder.

### 3.4. Stigma-related attitudes

Regarding stigma-related attitudes, students disagreed or strongly disagreed that individuals who go to counseling are mentally weak, individuals who go to counseling are crazy, individuals with mental health problems should handle problems on their own, individuals who go to counseling are unable to handle their own problems, individuals who go to counseling are lazy, and individuals who go to counseling are different from normal people in a negative way (Table 4).

**Table 4.** Stigma-related attitudes toward help-seeking for mental health problems.

I feel that individuals. …	*M*	SD
Who go to counseling for mental health problems are not able to solve their problems	1.71	0.779
Who go to counseling for mental health problems are mentally weak	1.66	0.772
With mental health problems should handle problems on their own without the help of counselors	1.64	0.786
Who go to counseling are different from normal people in a negative way	1.52	0.717
Who go to counseling for mental health problems are crazy	1.48	0.684
Who go to counseling for mental health problems are lazy	1.44	0.666

Note: *n* = 698; Means based on a 5-point Likert-type scale (1 = strongly disagree and 5 = strongly agree); and missing values are excluded.

MANOVAs were calculated to determine if stigma-related attitudes differed based on demographic and background characteristics. Results indicated that females were significantly less likely than males to hold stigma-related attitudes. Univariate *F*-tests identified specific subscale items that significantly differed. Females were less likely than males to perceive individuals who go to counseling as mentally weak, individuals who go to counseling as crazy, to feel that individuals with mental health problems should handle problems on their own, that individuals who go to counseling as not able to solve problems, that individuals who go to counseling are lazy, and to feel that individuals who go to counseling are different from normal people in a negative way. In addition, results found students with a family member or friend with a mental health disorder were significantly less likely to hold stigma-related attitudes than were students without a family member or friend with a mental health disorder. Univariate *F*-tests identified specific subscale items that significantly differed and found that those with a family member or friend with a mental health disorder were less likely than their counterparts to perceive individuals who go to counseling as mentally weak, individuals who go to counseling as crazy, that individuals who go to counseling as not able to solve problems, that individuals who go to counseling are lazy, and to feel that individuals who go to counseling are different from normal people in a negative way. Results indicated no significant differences in stigma-related attitudes based on grade, involvement in a campus organization, and race/ethnicity.

In addition, MANOVAs were conducted to determine if stigma-related attitudes differed based on perceived benefits and barriers. Results indicated that students perceiving lower benefits to treatment were significantly more likely to hold stigma-related attitudes *F*(1, 6) = .032, *p* = .001. Univariate *F*-tests were subsequently calculated and determined that students with low benefits were more likely to perceive individuals who go to counseling as mentally weak, crazy, unable to handle their own problems without help, unable to solve problems, lazy, and different from normal people in a negative way. Similarly, results indicated that students perceiving higher barriers to treatment were significantly more likely to hold stigma-related attitudes *F*(1, 6) = 0.038, *p* < .001.

Regarding mental health counseling, students ever receiving mental health counseling perceived significantly less stigma than those who had not received mental health counseling. Specifically, students receiving counseling were less likely to perceive individuals receiving counseling as mentally weak, as unable to solve their own problems, as lazy, and as different from normal people in a negative way (Table 5).

**Table 5.** Students' perceived stigma based on perceived benefits and barriers.

Stigma-related attitudes	Number of perceived benefits			Number of perceived barriers		
	*M* (SD)	*F*	*P*	*M* (SD)	*F*	*p*
I feel that individuals who go to counseling for mental health problems are mentally weak						
Low	1.79 (0.798)	15.573	<.001	1.73 (0.784)	5.724	.017
High	1.56 (0.738)			1.59 (0.756)		
I feel that individuals who go to counseling for mental health problems are crazy						
Low	1.58 (0.727)	10.595	.001	1.57 (0.722)	11.683	.001
High	1.41 (0.641)			1.40 (0.636)		
I feel that individuals with mental health problems should handle problems on their own without the help of counselors						
Low	1.73 (0.797)	6.199	.013	1.75 (0.831)	11.708	.001
High	1.58 (0.773)			1.54 (0.730)		
I feel that individuals who go to counseling for mental health problems are not able to solve problems						
Low	1.82 (0.799)	11.227	.001	1.80 (0.795)	9.202	.003
High	1.62 (0.754)			1.62 (0.756)		
I feel that individuals who go to counseling for mental health problems are lazy						
Low	1.56 (0.715)	15.441	<.001	1.54 (0.741)	14.457	<.001
High	1.35 (0.615)			1.35 (0.574)		
I feel that individuals who go to counseling are different from normal people in a negative way						
Low	1.65 (0.749)	15.992	<.001	1.66 (0.785)	23.675	<.001
High	1.43 (0.678)			1.35 (0.574)		

Note: *n* = 698; Means based on a 5-point Likert-type scale (1 = strongly disagree and 5 = strongly agree); number of perceived benefits dichotomized into high (11–14 perceived benefits) and low (0–10 perceived benefits) based on the median split; number of perceived barriers dichotomized into high (9–14 perceived barriers) and low (0–8 perceived barriers) based on the median split; and missing values are excluded.

## 4. Discussion

The present study examined benefits and barriers to help-seeking as well as stigma-related attitudes towards mental health treatment. Regarding perceived benefits, the most often reported benefits related to receiving mental health services were reduced feelings of stress, improved mental functioning, and resolution of one's problems. Thus, college students did appear to believe that receiving mental health services helped to resolve problems, perhaps indicating that many perceive treatment as effective. Several characteristics were found to be associated with perceived benefits and barriers of receiving mental health services as well as stigma-related attitudes toward mental health treatment.

Several study variables were found to differ based on sex. Results indicated that females were more likely to report benefits to participating in mental health services compared to males. This is consistent with the professional literature which indicates that females hold more positive attitudes toward mental health treatment than males (Chandra & Minkovitz, 2006). As females are more positive about services, they may be more likely to seek, enter, and remain in services than males. Similarly, study results revealed that females were significantly less likely than males to hold stigma-related attitudes. This is consistent with previous research which also found that males hold higher levels of perceived stigma than females (Chandra & Minkovitz, 2006).

Based on study findings, it is apparent that males may be less likely than females to seek treatment due to low perceived barriers as well as high stigma-related attitudes. Including these two variables as part of prevention and intervention strategies targeting male college students may be warranted. Campus-based mental health providers may educate male students on signs and symptoms of mental health disorders, when to seek treatment for mental health disorders, and helping others to seek treatment when needed. Public health messages sent through email, text message, and other social media channels aimed at encouraging males to participate in mental health services may increase males' positive attitudes toward treatment effectiveness, reduce stigma-related attitudes, and ultimately encourage males to participate in mental health services. Offering brief overviews of mental health treatment in classes, residence halls, and other key areas on campus may also heighten awareness and clarity surrounding mental health disorders. In addition, college health professionals may offer educational programs targeting males with information on the benefits of mental health treatment and the importance of seeking help when needed. All strategies should be evaluated with future research to determine the effect on college students, particularly males.

Contrary to general population studies which reveal that women are more likely to seek out mental health services compared to men (Haunstein et al., 2006; Mackenzie, Gekoski, & Knox, 2006), the present study found no significant differences in the number of perceived barriers to help-seeking behaviors based on sex. Several studies of college students found that male college students hold more negative perceptions and attitudes toward help-seeking behaviors than their female counterparts. Researchers speculate that this is primarily due to traditional social norms and gender roles that characterized males based on strength and lack of emotional expression (Addis & Mahalik, 2003; Ang, Lim, Tan, & Yau, 2004; Mojtabai, 2007). Overall, there have been mixed results among the college student population regarding sex differences (Rosenthal & Wilson, 2008). Thus, future studies should further explore sex differences regarding perceived barriers to help-seeking behaviors for mental health problems. Perhaps, exploring traditional gender roles or using qualitative research methods could help shed light on perceived benefits and barriers as well as stigma-related differences.

Regarding race/ethnicity, several differences were found. In contrast to our expectation, individuals in minority groups did not perceive more benefits to receiving mental health services than white youth. In fact, the opposite was true with white youth reporting higher levels of benefits for participating in mental health services than those in minority groups. This finding is inconsistent with existing literature as previous research has found that minorities perceive more positive attitudes towards mental health services than whites (Chamberlain et al., 2000; Gonzalez et al., 2005). In addition, white students were significantly more likely to perceive barriers to help-seeking than non-white students. Although previous general population studies suggest ethnic minorities report higher perceived barriers than white individuals, limited evidence exists for the college population (Kearney, Draper, & Baron, 2005; Masuda, Suzumura, Beauchamp, Howells, & Clay, 2005; Rosenthal & Wilson, 2008). An alternative explanation for this finding regarding race as a significant predictor of help-seeking behaviors among college students in the present study is based on cultural influences (Cauce et al., 2002). For instance, previous research has found that minority groups prefer to seek guidance for mental health problems from clergy and family more frequently than from professional, mental health resources (Ayalon & Young, 2005; Kane, 2010). Nevertheless, there are mixed results based on help-seeking attitudes among minority groups (Anglin, Alberti, Link, & Phelan, 2008; So, Gilbert, & Romero, 2005. Based on study findings, research examining differences based on race/ethnicity may warrant further exploration in future studies. More specifically, future studies should examine potential cultural influences on barriers to help-seeking behaviors among the college student population.

Concerning barriers to mental health treatment, the present study identified the top perceived barriers to mental health help-seeking behaviors among college students as “embarrassment”, “denial”, and “not wanting to be labeled as crazy”. Consistent with previous research, denial has been established as a barrier to help-seeking behaviors, given that individuals do not want to acknowledge their own mental health problems and generally hold negative attitudes about individuals who seek help (Corrigan, 2004). Perceived stigma may impact students' help-seeking behaviors by reducing communication about their mental health problems due to embarrassment and fear that others will label them for seeking help (Eisenberg, Downs, Golberstein, & Zivin, 2009). Thus, educating individuals about the importance of help-seeking and increasing comfort in discussing mental health with important others are important aspects of interventions aimed at decreasing stigma-related attitudes (Pinfold et al., 2003). Such information should be used when planning mental health interventions with college students.

The present study found that perceived barriers to help-seeking differed significantly by age and previous utilization of counseling services. Regarding age, the present study revealed that freshman and sophomore students were more likely to perceive a significantly greater number of barriers than junior, senior, and graduate students. This is not surprising since a considerable amount of previous research suggests that age is a predictive factor for help-seeking behaviors since younger individuals have more negative, stigmatizing attitudes toward mental health services than older individuals (Golberstein et al., 2009; Gonzalez et al., 2005; Ojeda & Bergstresser, 2008). Younger students tend to experience more symptoms of mental health problems typically related to academic transitions and social outcomes (Breslau, Lane, Sampson, & Kessler, 2008). Thus younger students may not know how to deal with these transitions and will avoid seeking help to resist being labeled as “crazy” than their older counterparts. Such findings underscore the importance of reducing barriers to help-seeking behaviors of students entering college by educating them about academic transitions, social strains, and by increasing the awareness of counseling services offered on college campuses.

Those who had previously received counseling reported more barriers to treatment than those who had not. This finding was unexpected and could highlight that those who had received counseling had a better idea of wait times and other “access” barriers that may make it difficult to begin treatment. Perhaps, participants who have received counseling view more barriers than participants who have not received counseling since seeking counseling services again could involve fear of self-disclosing personal information to a new counselor. Further research is warranted on how to increase counseling experiences as well as inform students about the private nature of counseling services.

The present study found that students held low overall stigma-related attitudes. Of the stigma-related attitudes, students were least likely to perceive individuals who go to counseling for mental health problems as crazy or lazy. Regarding differences, students ever receiving counseling were less likely to hold stigma-related attitudes than students who did not receive counseling. This is consistent with previous research that suggests that individuals never receiving treatment hold higher levels of stigma-related attitudes (Lally, O'Conghaile, Quigley, Bainbridge, & McDonald, 2013).

Regarding family and friends, experience with a family member's mental health treatment was related to participants' perceiving more benefits related to mental health treatment. This finding is consistent with previous studies, which found family history of mental health treatment to be positively associated with seeking counseling (Corrigan et al., 2001). In addition, students with family or friends with a mental health disorder were less likely than students without family or friends with a mental health disorder to hold stigma-related attitudes. Previous research revealed that adults having a family member with mental illness held lower stigma-related attitudes than their counterparts (Corrigan et al., 2001). Corrigan et al. (2001) found that adults who were more familiar with mental illness, through having a family member with mental illness, were less likely to endorse negative attitudes toward these people. Similarly, Eisenberg et al. (2011) reported that having friends and family members who had received treatment was related to participation in mental health treatment. These positive attitudes may be influenced by contact, as experience with people with mental health problems can be related to more positive attitudes toward them (Read & Law, 1999). As personal experience seems to be relevant, increasing college students' exposure to mental health issues and educating students on mental health may be methods of reducing stigma. Additional studies should evaluate the effectiveness of exposure and education on stigma-related attitudes.

### 4.1. Limitations

Several factors may have limited the generalizability of study findings. First, the survey's monothematic nature may have resulted in a response set bias. Second, as data were self-reported by participants, socially desirable responses may have been reported by some individuals. Third, as the sample was comprised of students from one Midwestern university, caution should be exercised in generalizing results to other populations. Fourth, ratings of stigma were low, indicating fairly positive ratings. In future studies, it may be beneficial to use qualitative methods to determine the basis for positive views as well as determine whether other variables, such as contact with peers who have benefited from mental health services, are influencing positive perceptions of those with mental health problems.
